# Genetic estimates of correlation and causality between blood-based biomarkers and psychiatric disorders

**DOI:** 10.1126/sciadv.abj8969

**Published:** 2022-04-06

**Authors:** William R. Reay, Dylan J. Kiltschewskij, Michael P. Geaghan, Joshua R. Atkins, Vaughan J. Carr, Melissa J. Green, Murray J. Cairns

**Affiliations:** 1 School of Biomedical Sciences and Pharmacy, The University of Newcastle, Callaghan, NSW, Australia.; 2 Centre for Brain and Mental Health Research, Hunter Medical Research Institute, Newcastle, NSW, Australia.; 3 School of Psychiatry, University of New South Wales, Randwick, NSW, Australia.; 4 Neuroscience Research Australia, Sydney, NSW, Australia.; 5 Department of Psychiatry, Monash University, Melbourne, VIC, Australia.

## Abstract

There is a long-standing interest in exploring the relationship between blood-based biomarkers and psychiatric disorders, despite their causal role being difficult to resolve in observational studies. In this study, we leverage genome-wide association study data for a large panel of heritable serum biochemical traits to refine our understanding of causal effect in biochemical-psychiatric trait pairings. We observed widespread positive and negative genetic correlation between psychiatric disorders and biochemical traits. Causal inference was then implemented to distinguish causation from correlation, with strong evidence that C-reactive protein (CRP) exerts a causal effect on psychiatric disorders. Notably, CRP demonstrated both protective and risk-increasing effects on different disorders. Multivariable models that conditioned CRP effects on interleukin-6 signaling and body mass index supported that the CRP-schizophrenia relationship was not driven by these factors. Collectively, these data suggest that there are shared pathways that influence both biochemical traits and psychiatric illness.

## Teaser

There was extensive genetic overlap between blood-based biochemical traits and psychiatric illness.

## INTRODUCTION

Psychiatric disorders arise from a complex interplay between genetic and environmental risk factors. Twin-based and genome-wide association studies’ (GWAS) heritability estimates have demonstrated the importance of genetic risk to the spectrum of psychiatric illness (*1*–*3*). In particular, GWAS has been successful in identifying regions of the genome associated with psychiatric disorders, as well as revealing both overlapping and distinct features among the genetic architecture of these traits (*2*–*6*). For example, our group previously demonstrated that several genes associated with schizophrenia (SZ) were shared with other psychiatric disorders, along with genes that appeared more specifically linked to SZ (*4*). The challenge for psychiatric genetics from here onward is to integrate and expand these data such that the biological insights gained may be directly relevant for psychiatric practice.

GWAS has proven valuable beyond just gene discovery in psychiatry, in which it allows the study of relationships between sets of traits in terms of genetic correlation (*7*), as well as GWAS informed methods for causal inference (*8*, *9*). An area of continued interest is the interplay between circulating biochemical factors and the pathophysiology of psychiatric disorders (*10*–*13*). These studies have endeavored to find biochemical traits readily detectable in blood, which, in theory, could be diagnostic or prognostic biomarkers for a given psychiatric disorder. Many of these hypotheses stem from the idea that peripheral biochemical traits may exert an effect on the brain, directly or indirectly through their effect on other mediators, and that the manifestation of mental illness is, in part, attributed to these factors that primarily act in the periphery (*11*, *12*, *14*–*16*). Identifying these biochemical-psychiatric relationships would be clinically valuable, as many of these traits can be modulated by existing drugs and/or lifestyle interventions. However, progress in this field has been hampered by small sample size studies, along with the fact that most of these studies are observational in nature and, thus, likely subject to at least some confounding. Genetics offers an attractive prospect for studying biochemical traits in psychiatry as many such measures are heavily influenced by genetic factors, with germline genetic variants fixed at birth and immune to reverse causation in most instances. These features of biochemical-associated germline variants can facilitate less-confounded estimates of correlations between traits, as well as provide potential instrumental variables (IVs) for causal inference approaches such as Mendelian randomization (MR) (*7*, *8*). In this study, we attempt to harmonize interstudy variability by using a panel of large sample size (*N* > 300,000) biochemical GWAS from a single cohort [UK Biobank (UKBB)] to investigate genetic overlap with different psychiatric disorders, along with putative causal effects. We found that the majority of biochemical traits tested were genetically correlated with at least one psychiatric trait, with evidence of convergent and divergent correlation profiles among the different disorders. We also demonstrated evidence that there may be a causal relationship on psychiatric phenotypes through circulating C-reactive protein (CRP), glucose, and urate, which may have direct implications for clinical practice.

## RESULTS

### Widespread genetic correlation between blood-based biomarkers and psychiatric traits

**Fig. 1.**
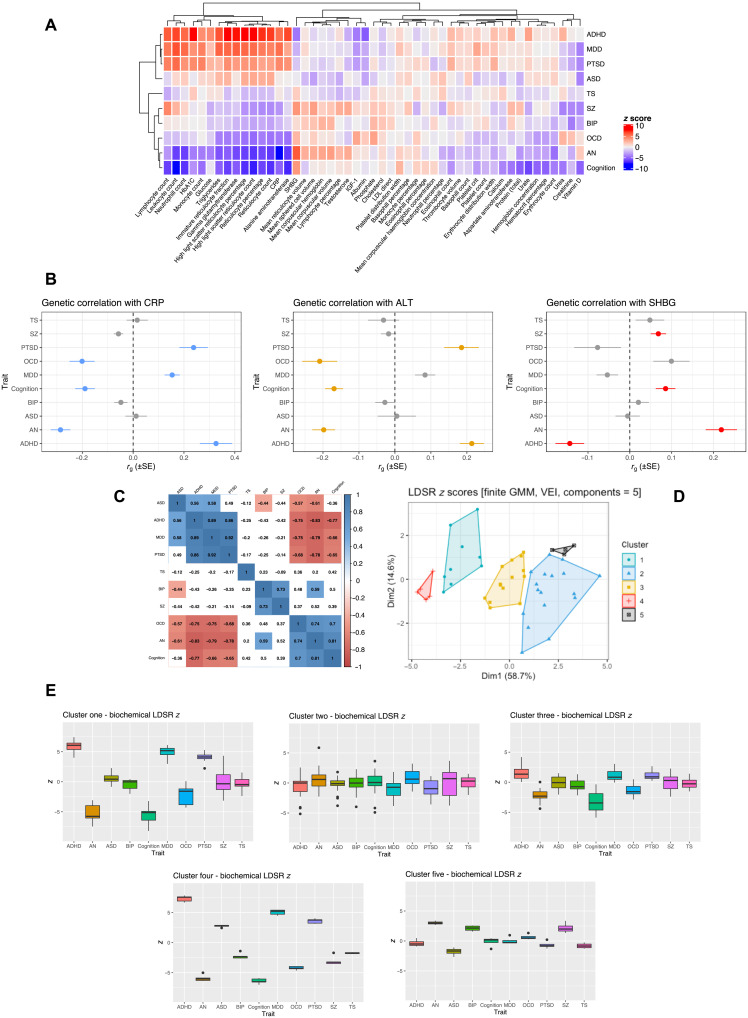
Genetic correlation between blood-based biomarkers and psychiatric GWAS. (**A**) Heatmap of LDSR correlation *z* scores (*r*_g_/SE) between each psychiatric trait and 49 biochemical GWAS. The psychiatric and biochemical traits are grouped on the *x* and *y* axes, respectively, by hierachial clustering using Pearson’s distance. (**B**) Examples of biochemical traits with evidence of discordant genetic correlations among the different psychiatric phenotypes. CRP, alanine aminotransferase (ALT), and sex hormone binding globulin (SHBG) are presented for illustration. The forest plot denotes the LDSR *r*_g_, with its SE representing the confidence bars. Traits highlighted in blue, orange, and red for CRP, ALT, and SHBG, respectively, were significantly correlated after the application of multiple testing correction. (**C**) Correlation matrix (Pearson) of LDSR *z* score between each trait, correlation estimates that survive correction for the number of tests performed are highlighted. (**D**) Components of biochemical LDSR *z* scores derived using finite Gaussian mixture modeling (GMM)—the optimal parametrization of the variance-covariance matrix was five components with diagonal distribution, variable volume, and equal shape (VEI). The components are plotted relative to their contribution to the first and second principal components of the LDSR *z* score matrix. (**E**) Box-and-whisker plots of the LDSR *z* scores for each disorder composed of traits assigned to each of the five components derived from the GMM procedure.

We tested the genetic correlation between a panel of blood-based biomarkers from the UKBB and 10 psychiatric GWAS using linkage disequilibrium score regression (LDSR). We found that 61% (*N* = 30) of the biochemical traits tested were significantly correlated with at least one psychiatric trait after multiple testing correction, with every psychiatric trait exhibiting a significantly nonzero biochemical correlation after correction, except for Tourette’s syndrome (TS) (Fig. 1A and tables S2 to S11). The most significantly correlated biomarker for each trait is outlined in Table 1.

**Table 1.** The most significant genetic correlation between each trait and a biochemical GWAS.

**Biochemical trait**	Psychiatric trait*	***r*_g_^†^**	**SE**	***P***
High light scatter reticulocyte percentage	ADHD	0.256	0.033	4.58 × 10^–15^
High light scatter reticulocyte count	OCD	−0.212	0.046	3.37 × 10^−6^
CRP	AN	−0.286	0.038	9.76 × 10^–14^
Vitamin D	ASD	−0.177	0.047	2 × 10^–4^
Creatinine	BIP	−0.106	0.027	7.23 × 10^–5^
Leukocyte count	Cognition	−0.198	0.024	2.66 × 10^–16^
Leukocyte count	MDD	0.155	0.025	9.77 × 10^–10^
Leukocyte count	PTSD	0.225	0.043	1.37 × 10^–7^
Lymphocyte count	SZ	0.075	0.017	1.52 × 10^–5^
Lymphocyte count	TS	−0.093	0.039	0.017

*Psychiatric GWAS: attention-deficit/hyperactivity disorder (ADHD), anorexia nervosa (AN), autism spectrum disorder (ASD), bipolar disorder (BIP), major depressive disorder (MDD), obsessive-compulsive disorder (OCD), post-traumatic stress disorder (PTSD), SZ, and TS.

†*r*_g_ is the estimate of genetic correlation from LDSR.

There was clear evidence of biomarkers with divergent genetic correlations between different psychiatric phenotypes, for instance, CRP, alanine aminotransferase (ALT), and sex hormone binding globulin (SHBG; Fig. 1B). In the case of CRP, it displayed a negative correlation after correction with obsessive-compulsive disorder (OCD), anorexia nervosa (AN), and cognition, as well as a trend toward a negative correlation with SZ (*P* = 1.7 × 10^−3^), while its correlation with post-traumatic stress disorder (PTSD), major depressive disorder (MDD), and attention-deficit/hyperactivity disorder (ADHD) was strongly positive. These data provide some support to recent cohort studies, including lower CRP observed in patients with eating disorder (*17*), while elevated CRP was associated with ADHD (*18*). Notably, this contradicts previous observational estimates of elevated CRP in SZ (*19*). Moreover, there were 14 biochemical traits that were only correlated after multiple testing correction with two or fewer psychiatric GWAS. Some examples of biochemical traits correlated with two or fewer psychiatric GWAS were albumin and ADHD (*r*_g_ = −0.151), mean corpuscular volume and AN (*r*_g_ = 0.087), mean sphered cell volume and SZ (*r*_g_ = 0.06), and creatinine with bipolar disorder (BIP) and SZ (SZ: *r*_g_ = −0.07; BIP: *r*_g_ = −0.106).

We further investigated the relationship between the profiles of 49 LDSR biochemical *z* scores for each psychiatric trait. We observed strong positive and negative correlations between the trait-wise LDSR biochemical *z* score for each trait, which we term the biochemical correlation profile (Fig. 1C). For instance, the ADHD biochemical correlation profile demonstrates large-magnitude positive correlations with autism spectrum disorder (ASD), MDD, and PTSD but negative correlations with OCD, AN, and cognition. This can be interpreted as traits that tend to be positively correlated with ADHD are also positively correlated with ASD, MDD, and PTSD and vice versa for OCD, AN, and cognition. These relationships were further interrogated by subjecting the 10 biochemical correlation profiles to finite Gaussian mixture modeling (GMM; Fig. 1D). We observed five components (clusters) with diagonal distribution, variable volume, and equal shape as the most parsimonious parameterization of the covariance matrix (table S12). The biochemical correlation profile LDSR *z* scores within each cluster are plotted in Fig. 1E. Briefly, the first component was composed of a series of traits with discordant correlations between the disorders, such as CRP, ALT, and glycated hemoglobin (HbA1c), while the second and third components were a diverse set of biomarkers with more similar LDSR *z* scores across the psychiatric disorders tested. Component four was notable as it was solely composed of reticulocyte (immature erythrocytes)–related traits, which, analogous to component one, was quite discordant in its correlation profiles. The fifth and final component was composed of other erythrocytic-related traits; however, the differences between disorders were less marked than component four. Together, this demonstrates that groups of biomarkers tend to have similar relationships with different psychiatric traits.

### Genetically proxied biochemical measures were associated with a severe cognitive deficit schizophrenia subtype

**Fig. 2.**
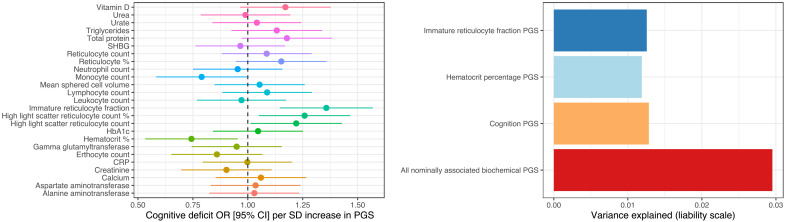
The association between biochemical PGS and a severe CD subtype of SZ. Left: Forest plot of the association (OR with 95% CI) between an SD increase in each PGS and severe CD, relative to SZ cases with less marked impairment (CS). Right: Variance explained on the liability scale, assuming a 0.33% CD population prevalence for biochemical and a general cognitive ability PGS.

We tested the association between 25 genetically proxied biomarkers [polygenic scores (PGS), constructed from the UKBB GWAS] that were correlated with either the SZ or the general cognitive ability GWAS with a severe cognitive deficit (CD) subtype of SZ relative to a subset of cases with less-marked impairment [cognitively spared (CS)] from the Australian Schizophrenia Research Bank (ASRB) cohort (*N* = 391). As cognitive performance is highly variable among SZ cases, a multidimensional grade of membership (GoM) clustering of nine cognitive measures was previously applied to derive these two subgroups of cognitive performance in the ASRB (*20*). There were two biochemical PGS that displayed a relatively significant association with CD status—hematocrit percentage and immature reticulocyte faction (Fig. 2 and table S13). Each SD increase in the immature reticulocyte fraction PGS was associated with a 35.7% [95% confidence interval (CI): 14.5%, 56.9%; *P* = 4.72 × 10^−3^, *q* = 0.07] increase in the odds of severe CD. Conversely, genetically proxied hematocrit percentage exerted a protective effect: odds ratio (OR) = 0.744 [95% CI: 0.533, 0.954], *P* = 5.82 × 10^−3^, *q* = 0.07. We emphasize that these signals only survive multiple testing correction using a lenient false discovery rate (FDR) threshold of 10%; however, given the small sample size of this cohort, we believe that these findings remain noteworthy. Immature reticulocyte fraction was negatively correlated with cognition, with a trend toward a negative relationship with SZ as well. Hematocrit percentage was also negatively correlated with cognition; however, there was a depletion of hematocrit percentage alleles among CD versus CS SZ cases, suggesting that a more complex relationship may be present. Both the hematocrit and reticulocyte fraction PGS explained around 1% of phenotypic variance in CD on the liability scale, which is similar to a PGS for general cognitive ability (Fig. 2). A model constructed with all nominally CD-associated PGS (*P* < 0.05) explained almost 3% in phenotypic variance. There was a nonsignificant trend of enrichment of SZ polygenic risk score (PRS) (*P* = 0.054) in CD. While these values are modest, it does suggest that the genetic architecture of biochemical traits is correlated with the severity of cognitive impairment in SZ. In addition, these estimates of phenotypic variance explained are likely inflated relative to that of an external sample, and, thus, future investigation of the relationship between these PGS and cognition in SZ is warranted to test the replicability of these findings.

### Strong evidence of partial genetic causality between blood-based biomarkers and neuropsychiatric illness

A latent causal variable (LCV) model was constructed between each significantly correlated biochemical-psychiatric trait pair (UKBB biochemical GWAS and psychiatric GWAS) to estimate partial genetic causality (*9*). This approach uses genome-wide single-nucleotide polymorphism (SNP) effect estimates for each trait to evaluate evidence whether the effect of one trait on the second is larger than the reverse direction. Partial genetic causality is expressed by this framework as the posterior mean genetic causality proportion ($\hat{\text{GCP}}$), with |$\hat{\text{GCP}}$| > 0.6 considered as strong evidence of partial genetic causality and its sign related to which trait is postulated as partially genetically causal for the other (Materials and Methods). We can then also infer the consequence of the partial genetic causality of one trait on another using the sign of the genetic correlation. There were five instances where we found strong evidence of a potential causal relationship, with all of them suggesting an effect of the biochemical measure on the psychiatric trait rather than vice versa (Table 2 and table S14). These were as follows: glucose on ADHD ($\hat{\text{GCP}}$ = 0.64), CRP on AN ($\hat{\text{GCP}}$ = 0.92), urate on cognition ($\hat{\text{GCP}}$ = 0.88), CRP on MDD ($\hat{\text{GCP}}$ = 0.62), and CRP on OCD ($\hat{\text{GCP}}$ = 0.75). It is important to emphasize that these posterior mean $\hat{\text{GCP}}$ are not magnitudes of causal effect and only imply that there is a causal relationship between the biochemical traits and the psychiatric phenotype. Given the sign of the genetic correlation, we can likely infer that glucose may increase the risk of ADHD and urate could have a deleterious effect on general cognitive function. As outlined in a previous section, CRP has highly divergent correlations, and these data coupled with the LDSR further support a protective effect on AN and OCD, while it is likely risk increasing for MDD. SZ did not quite survive multiple testing correction for a genetic correlation with CRP; however, given previous evidence of a protective effect of CRP on SZ from MR studies (*21*–*23*), we also constructed an LCV model between CRP and SZ and found moderate support for this relationship ($\hat{\text{GCP}}$ = 0.56, SE = 0.23, *P* = 5.11 × 10^−6^). Given that the genetic correlation is only small, it is less likely that previous MR studies were unduly biased by genetic correlation. In addition, we also observed an unusual phenomenon in the HbA1c and PTSD model, whereby the posterior mean $\hat{\text{GCP}}$ was strongly positive ($\hat{\text{GCP}}$ = 0.76), implying an effect of HbA1c on PTSD, while its *z* score was negative (*z* = −6.51), which signifies the opposite. As described in the Supplementary Materials and fig. S1, we found that these conflicting data were likely attributable to a rare violation of the LCV model assumptions, whereby the mixed fourth moments had opposite signs to each other and the genetic correlation. This could be explained by certain SNPs having highly divergent effects from the rest of the genome-wide signal. Moreover, there were two other trait pairs that trended toward partial genetic causation (|$\hat{\text{GCP}}$| > 0.5) but did not exceed the stringent 0.6 threshold. These were SHBG on cognition ($\hat{\text{GCP}}$ = 0.55) and triglycerides on OCD ($\hat{\text{GCP}}$ = 0.55). We then sought to replicate the LCV findings by using different previously published GWAS for glucose, urate, CRP, and HbA1c. Despite smaller sample sizes, we found relatively consistent GCP estimates that supported the above models using the UKBB GWAS (table S15). Specifically, CRP → AN and CRP → OCD still obtained $\hat{\text{GCP}}$ > 0.6, while CRP → MDD, urate → cognition, and glucose → ADHD still trended toward strong evidence of partial genetic causality using these smaller biochemical GWAS ($\hat{\text{GCP}}$ > 0.4). However, HbA1c did not show strong evidence of partial genetic causality ($\hat{\text{GCP}}$ = 0.13) on PTSD using a smaller sample size GWAS from Wheeler *et al.* (*24*).

**Table 2.** Strong evidence of partial genetic causality of a biochemical measure on a psychiatric trait.

**Biochemical trait**	Psychiatric trait*	**GCP^†^**	**SE**	***P* ^‡^**	***r*_g_**
Glucose	ADHD	0.64	0.28	0.035	0.134
CRP	AN	0.92	0.07	5.95 × 10^–56^	−0.286
CRP	MDD	0.62	0.21	1.57 × 10^–12^	0.154
CRP	OCD	0.75	0.16	2.07 × 10^–18^	−0.201
Urate	Cognition	0.88	0.09	5.27 × 10^–114^	−0.1047

*Psychiatric GWAS: ADHD, AN, MDD, and OCD.

†The posterior mean GCP with its accompanying posterior SE in the adjacent column.

‡*P* value that tests whether the GCP estimate is significantly different from zero.

### C-reactive levels exert a direct protective effect on schizophrenia conditioned on body mass index and interleukin-6 signaling

**Fig. 3.**
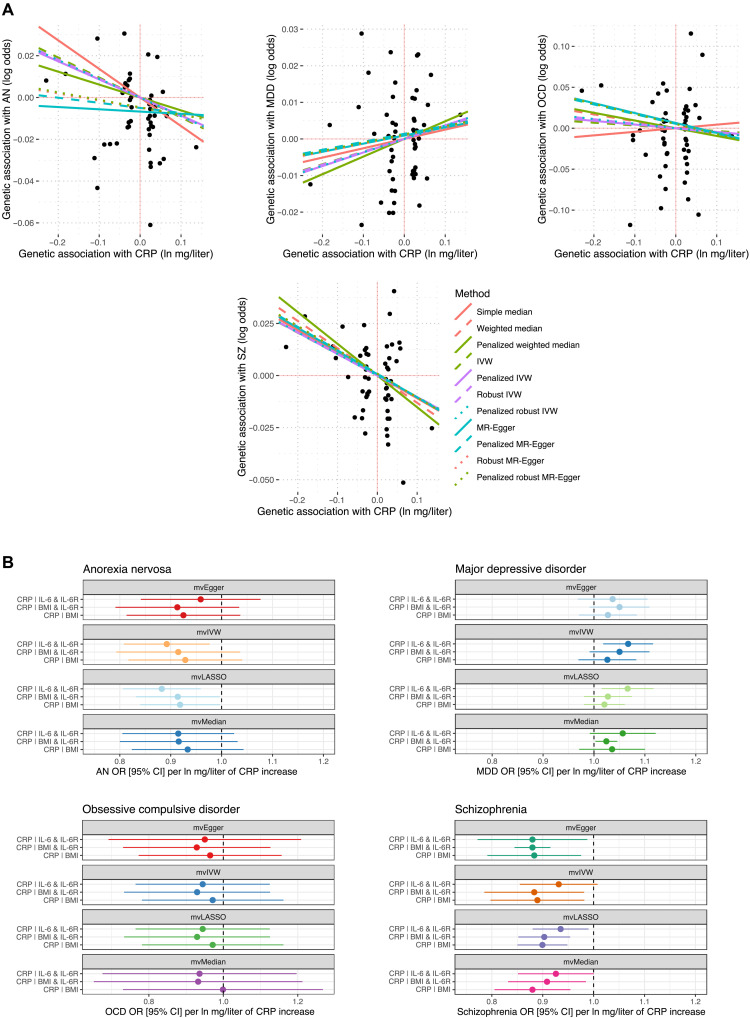
Total and direct estimated effect of CRP on psychiatric illness. (**A**) Total effect of CRP on each psychiatric outcome considered. Each point represents the IV-exposure effect versus the IV-outcome effect. Trend lines are indicative of the slope of each MR method used. The outcomes plotted from left to right are AN, MDD, OCD, and SZ. (**B**) Direct effect of CRP in MVMR models—the three models contained the following phenotypes as additional exposures: circulating interleukin-6 (IL-6) and its receptor (IL-6R), body mass index (BMI), and IL-6R and BMI.

CRP displayed strong evidence of partial genetic causality on three psychiatric disorders, and, thus, we sought to further analyze these relationships by estimating the total effects and direct impact of CRP using univariable MR and multivariable MR (MVMR), respectively (tables S16 to S22). We included SZ in these analyses, due to evidence from previous SZ GWAS that CRP exerts a protective effect on SZ liability, as discussed in the previous section. MR methods differ from the LCV approach in that they leverage specific independent SNPs strongly associated with trait one (the exposure trait) as IVs to estimate a causal effect size of the exposure trait on the outcome, given that a variety of assumptions are met (Materials and Methods). MR estimates can be biased by genetic correlation, which is explicitly modeled by the LCV approach, but they have the advantage of facilitating direct causal estimates and the ability to construct multivariable models (*8*, *9*, *25*). The CRP GWAS used here was drawn from a non-UKBB cohort such that there was no sample overlap with the AN and MDD GWAS (Supplementary Materials) (*26*). Using our primary model [inverse-variance weighted (IVW) estimator with multiplicative random effects], we found evidence that a natural log-transformed milligrams per liter increase in CRP was associated with a statistically significant reduction in the odds of SZ (OR = 0.91 [95% CI: 0.85, 0.98], *P* = 0.01) and AN (OR = 0.91 [95% CI: 0.83, 0.99], *P* = 0.03), which supports the LDSR-inferred direction of the LCV relationship. Using a less-conservative IVW estimator with fixed effects yielded a more precise estimate in both instances, as expected (CRP → SZ: *P* = 6.29 × 10^−5^; CRP → AN: *P* = 9.79 × 10^−3^). There was a trend toward an odds-increasing effect of CRP on MDD (*P* = 0.19), while there was no indication of a reliable effect in the OCD model (*P* = 0.82). A nonsignificant estimate from MR does not necessarily preclude the existence of a causal relationship supported by an LCV model, although LCV models with corresponding MR support would perhaps be viewed as stronger evidence. We discuss the sensitivity analyses for each of these univariable models in detail in Supplementary Text. Briefly, the five MR tests deployed with different assumptions regarding IV validity [plurality valid, majority valid, and instrument strength independent of direct effect (InSIDE) assumption] had very similar point estimates (OR range: 0.88 to 0.91) and were all statistically significant for CRP → SZ with the exception of the simple median (*P* = 0.07). The CRP → AN estimate across the different models were also directionally consistent; however, they were not statistically significant (except for some contamination mixture models with different prespecified SD of invalid IVs), and, thus, the total effect of CRP on AN by MR has comparatively weaker evidence compared to SZ. The effect of using robust, penalized, or robust penalized weights in the median, IVW, and Egger models was not marked for each of the CRP to psychiatric models (Fig. 3A and table S21). In the CRP → SZ and CRP → AN models, the Egger intercept was not significantly different from zero (although there was a trend in the AN model; *P* = 0.07), and, thus, there was no strong statistical evidence of confounding pleiotropy using this metric. However, there was evidence of heterogeneity between the IV ratio estimates for AN and SZ: The MR-PRESSO global test of pleiotropy was significant and Cochran’s *Q* statistic was significant for AN and SZ, although both causal estimates remained statistically significant in the MR-PRESSO outlier–corrected estimates (table S19). Given the biological complexity of these phenotypes, heterogeneity does not necessarily imply confounding pleiotropy. Moreover, using a leave-one-out analysis, we found evidence of one outlier IV in the SZ model, while there were three outlier SNPs in the AN model (table S20), although the effects of removing these IVs were relatively small. The outlier IV in the SZ model was also proximal to the CRP gene itself (IVW *P* = 0.07 when removed), meaning that it is likely to influence SZ through CRP rather than being indicative of confounding pleiotropy. Specifically, the IV used was the lead SNP for the locus mapped to the *CRP* gene in the GWAS we used and is in high LD with putative functional variants within the 3′ untranslated region of *CRP*. Given that the CRP estimates on AN and SZ remained consistent upon removing outliers by MR-PRESSO or through iterative single IV exclusion, it is less likely that horizontal pleiotropy fully explains these signals. There was also no evidence using a reverse MR model that, with the psychiatric disorder as the exposure, there were bidirectional effects, although these models are inherently underpowered and are best treated as a test of the null hypothesis only (table S22).

We sought to investigate the direct effect of circulating CRP on the above psychiatric phenotypes using MVMR conditioning on interleukin-6 (IL-6) signaling and body mass index (BMI; Materials and Methods). These variables were selected as IL-6 signaling is the key upstream factor that stimulates hepatic CRP secretion, and, thus, genetic IVs for CRP could plausibly operate through IL-6 to independently influence the risk of psychiatric illness (*27*). Moreover, BMI is postulated to be associated with CRP biology and may act upstream or even as a mediator of CRP-related effects (*28*, *29*). We found that CRP exhibited a robust direct protective effect on SZ conditioned on IL-6 signaling and BMI (table S23 and Fig. 3B). For instance, using a multivariable IVW estimator, the direct effect of CRP on SZ conditioned on BMI and IL-6 receptor (IL-6R) was analogous to the univariable IVW total estimate (OR = 0.88 [95% CI: 0.79, 0.98], *P* = 0.01). There was no evidence of an effect of BMI or IL-6 signaling on SZ conditioned on CRP. Similarly, the effect size of the CRP → AN MVMR model remained similar to that estimated in the univariable constructs (Fig. 3B); however, these estimates were only statistically significant in a subset of the models (table S23). As a result, the MR evidence for the direct protective effect of CRP on AN is comparatively weaker than the CRP → SZ model, as was seen in the univariable estimates. We also observed some evidence to suggest that IL-6 abundance exerts a protective effect on AN conditional on CRP and IL-6R, which was nominally nonzero in every MVMR model except for the median estimator (OR = 0.96 [95% CI: 0.93, 0.99] per unit increase in IL-6, *P* = 0.037; multivariable IVW with multiplicative random effects). While evidence for a direct effect of CRP on MDD conditioned on each variable set was weak (Fig. 3B and table S23), there was consistent evidence that elevated IL-6R was associated with increased odds of MDD conditioned on CRP and IL-6 or CRP and BMI. For example, each unit increase in blood IL-6R protein expression was estimated to increase the odds of MDD by 2.7% [95% CI: 0.6%, 4.9%], conditioned on CRP and BMI. Last, there was no evidence in the MVMR models to support the protective effect of CRP on OCD; however, there was evidence that increased BMI decreases the odds of OCD conditioned on CRP and IL-6 signaling (table S23). This BMI → OCD effect estimate was quite large, albeit with wide CIs—OCD OR per SD increase in BMI conditioned on CRP and IL-6R = 0.52 [95% CI: 0.14, 0.91], *P* = 1 × 10^−3^.

### C-reactive protein displays overlapping association signals with schizophrenia and may have downstream impacts on the brain

There were five SZ GWAS lead SNPs (*P* < 5 × 10^−8^) that also obtained genome-wide significance in the UKBB CRP GWAS (table S24, Supplementary Text, and fig. S2). The signals were investigated using colocalization as described more extensively in Supplementary Text. For three of these loci, colocalization analyses using the European-only subset of the SZ GWAS and default prior probabilities demonstrated strong evidence for the association of three of these loci with both SZ and CRP; however, there was likely a different underlying causal variant (table S24). In other words, the posterior probability (PP) for the hypothesis termed *H*_3_ (locus associated with both traits but different underlying causal variant) was greater than 80% (table S24). These estimates remained consistent using larger prior probabilities for the hypothesis of a shared causal variant (*H*_4_) as a sensitivity analysis that is visualized in fig. S2. The remaining two loci also did not exhibit strong evidence of colocalization (denoting a shared causal variant; PP_*H*4_ > 0.8) or any of the other tested hypotheses (PP > 0.8). Local genetic correlation estimates with ρ-Heritability Estimation from Summary Statistics (HESS) demonstrated that 13 of the LD block partitions of the human genome displayed nonzero local covariance between CRP and SZ (table S25 and fig. S3) after Bonferroni correction. Five of these LD blocks with strong evidence of local genetic covariance were positive, and, thus, for these regions of the genome, SZ and CRP were positively correlated in contrast to the genome-wide estimate of nominal negative correlation.

We also sought to investigate the downstream consequences of raised CRP. We estimated the effect of raised CRP on the expression of 3284 proteins in blood using univariable MR. Using a liberal FDR cutoff of 10%, we found that 95 proteins were putatively causally influenced by elevated CRP, with 45 of these proteins surviving a stricter FDR threshold of 5% (table S26). We emphasize that these analyses are exploratory in nature, and we treat the effect sizes of the CRP effect on each protein largely as a test of the null hypothesis that the two are not associated. The vast majority of proteins that prioritized using the IVW estimator were directionally consistent in the median, mode, and Egger sensitivity analyses (table S27). However, 20 of the FDR < 0.1 proteins were suggested to act in the opposite direction and causally influence CRP, as the estimated variance explained by the IVs was significantly larger in the outcome than the CRP exposure, although this does not rule out bidirectional effects (table S28). We found that these proteins putatively influenced by CRP were enriched in several pathways including glycemic signaling and lymphocyte biology (tables S29 to S31). Notably, there were neuronal pathways overrepresented with CRP-associated proteins, including axon guidance, dopaminergic synapse, neurogenesis, glial cell differentiation, and cholinergic synapse. For example, dopaminergic and cholinergic synapse overrepresentations were driven by the three genes (*AKT1*, *AKT2*, and *AKT3*) that encode the RAC-alpha/beta/gamma serine/threonine-protein kinase complex, which was measured as a single entity in the protein study. Collectively, these data provide preliminary evidence that CRP may influence the expression of neuronally relevant proteins, and subsequent studies should seek to investigate these associations and their significance for different psychiatric phenotypes.

## DISCUSSION

In this study, we investigated genetic correlation and causality between a diverse panel of biochemical traits and psychiatric disorders, as well as general cognitive ability and a cognitive deficit subtype of SZ. Our data demonstrated that there is clear evidence of genetic overlap between blood-based measures and psychiatric phenotypes, as quantified by LDSR, which may indicate shared variants and pathways that predispose to these traits. It should be noted that the pleiotropic effect of variants on other traits could mediate these relationships (*7*), while some of these trait pairings were further shown in this study to be consistent with evidence of causality rather than just correlation. The distribution of biochemical-psychiatric correlations demonstrated traits that often exhibited highly divergent correlations (different signs), as well as clusters of biochemical measures that tended to have similar psychiatric correlation profiles. For instance, we found that five reticulocyte traits clustered together (Fig. 1E), and they tended to have opposing psychiatric correlations (strong positive correlation with ADHD, MDD, and PTSD, while negative correlations with AN, OCD, SZ, and cognition). The genetic architecture of reticulocyte-related traits remains relatively uncharacterized; however, it is a highly polygenic trait that also has demonstrated genome-wide significant associations with rare nonsynonymous variation in genes such as *SPTA1*, *E2F4*, and *IFRD2* (*30*, *31*). Future studies should further investigate how the genetic factors that contribute to reticulocyte biology may also influence psychiatric traits. Given that the LCV posterior mean GCP estimates between the genetically correlated reticulocyte traits and each psychiatric phenotype were low, it suggests the existence of horizontal rather than vertical pleiotropy. We refer to horizontal pleiotropy as variants affecting both traits in a manner whereby their impact on one trait is not mediated by that of the other. In contrast, vertical pleiotropy denotes variants that causally link the traits in question (*32*).

A key advantage of our study is that we extended the findings from the LDSR models to estimate which biochemical-psychiatric trait pairs may represent causal relationships. We caution that all of these findings require validation in well-powered, replicated, randomized controlled trials to confirm that the causal effects do indeed exist. Trials for these kinds of circulating measures can be difficult to conduct due to their transient nature and the likely extended length of follow-up required to detect any effect on risk, further emphasizing the importance of genetic approaches. The putative effect of urate and glucose on cognition and ADHD, respectively, may have direct implications for drug repurposing given that compounds that modulate these traits are readily available. Both of these relationships are also supported by previous observational data (*33*–*35*). Data from an incident cohort study that treated individuals with urate-lowering compounds allopurinol and febuxostat demonstrated evidence of a risk-decreasing effect on dementia, supporting the deleterious effect of urate on general cognitive function (*36*).

The inferred effect of CRP on different psychiatric disorders was particularly interesting given that there was evidence of an odds-decreasing effect on AN, OCD, and SZ, while the opposite is true for MDD. CRP is traditionally conceptualized as a biomarker of chronic inflammation; however, its biology is likely somewhat more complex given that it is also implicated to play a direct role in pathogen response (*37*, *38*). Moreover, as reviewed by Del Giudice and Gangestad (*27*), CRP in its hepatically secreted pentameric isoform demonstrates some anti-inflammatory effects and may be a marker of other noninflammatory states. In our study, we also provide evidence that CRP levels may also exert an effect on proteins with neurological significance, including proteins enriched in pathways relevant to psychiatric illness such as axon guidance. Moreover, we show the previously documented protective effect of CRP on SZ through MR is not likely attributable to the effect of BMI and IL-6 signaling, which are closely related variables to CRP. Data from our study and previous examinations of the relationship between CRP and SZ through MR seemingly contradict previous observational evidence that CRP is elevated in SZ (*19*, *39*). If we assume that there is a causal effect, then there are a number of explanations that could account for this, although all require further investigation. First, previous observational studies that directly measured CRP in case/control cohorts could be confounded because of a variety of variables including lifestyle and general health or could even be caused by factors related to psychosis and/or SZ itself. While there was no evidence in this study using the LCV model or reverse MR that SZ causally influences CRP levels, local significant estimates of positive and negative genetic correlation between SZ and CRP observed for several LD blocks suggest a complex interrelationship between these phenotypes may exist that warrants additional study. Second, CRP itself may influence factors that affect the brain, directly or indirectly, and these factors may play a role in the pathogenesis of psychiatric illness. For instance, CRP was postulated in this study to causally up-regulate the expression of the RAC-alpha/beta/gamma serine/threonine-protein kinase complex (*AKT1*, *AKT2*, and *AKT3*), with impaired signaling by these serine/threonine kinases implicated to dysregulate dopaminergic neurotransmission and down-regulation of genetically predicted neuronal *AKT3* expression associated with SZ via a transcriptome-wide association study (*40*, *41*). Last, given that infection has been associated with liability of SZ, the role of CRP in pathogen defense may contribute to its putative protective properties. Further study on the neurobiological consequences of CRP signaling and its role in SZ is warranted, particularly to reconcile the discrepancies between observational studies and MR.

The putative causal effect of CRP on AN that was demonstrated in this study is, to our knowledge, a previously unidentified finding; however, it does support data from a recent longitudinal study that demonstrated that elevated CRP was associated with a protective effect on eating disorders (*17*), along with decreased measured CRP observed specifically in AN (*42*). It should be noted that, while the LCV data supported strong partial genetic causality of CRP on AN, the MR evidence was less statistically significant, with some evidence in the MVMR that IL-6 signaling may exert a protective effect on AN conditioned on CRP. Inhibition of the IL-6 pathway has been associated with weight gain, which may be protective for AN (*43*). Furthermore, CRP likely is intertwined with other metabolic factors, including insulin signaling, which our group has previously shown through MR is also putatively protective for AN (*44*). In MDD, we found some evidence to support that up-regulation of the IL-6R may be risk increasing conditioned on CRP and BMI, supporting preliminary data that blockade of IL-6R by agents such as tocilizumab may decrease depressive symptoms (*45*). However, data related to the antidepressant qualities of tocilizumab are conflicting (*46*), and randomized control trials are warranted to further investigate repurposing opportunities for IL-6 inhibition in MDD. Two recent studies also considered the effect of IL-6R signaling on the odds of depression via MR using some different GWAS than ours in some instances, with one study supporting a risk-increasing relationship between elevated IL-6R and depression (*47*), while the other suggested that IL-6R could be more specifically linked to suicidality rather than broad depression (*48*). Last, BMI demonstrated a quite robust protective effect on OCD conditioned on CRP and IL-6 signaling, in accordance with observational data that OCD is associated with reduced odds of obesity (*49*).

There are a number of important limitations that are central to the interpretation of the data in this study. Genetic correlations from LDSR likely reflect a shared underlying genetic architecture; however, this could be mediated by the relationship of the same genetic variants to another variable or variables (*7*). Despite this limitation, the existence of genetic correlation between traits is still informative as identifying genes, which affect both psychiatric and biochemical traits, and further insight into the mechanisms would likely refine our understanding of both traits. Moreover, the genetically informed causal inference approaches we implement in this study are subject to limitations regarding the data they are performed with and any biases therein, including potential effects of population stratification (*50*), selection bias (*51*), and the assumption of acyclicity, which refers to the lack of feedback loops between the exposure and outcome (*52*). The LCV model is also fixed to be bivariate in nature, and, thus, the effects of multiple meditators cannot be taken into account. We address this by constructing MVMR models such that direct effects are estimated, conditioned on likely confounders; however, our selection of confounders is not exhaustive, and other unidentified factors may influence our findings. The UKBB sample is also composed of middle-aged to older individuals over the age of 40, and, thus, more developmentally sensitive effects on the biochemical traits in question could not be assessed. Genetic variants are also sometimes claimed to represent a lifetime effect on a particular variable, although caution is required in this inference given that factors such as age may modulate the effect of a variant (*53*, *54*). We assert that, although GWAS-informed causal inference has a number of caveats and limitations, it enables an important opportunity to prioritize biochemical traits that are putatively clinically relevant in psychiatry and to inform future study into these traits.

## MATERIALS AND METHODS

### Study design

The principal aim of this study was a hypothesis-free test of genetic correlation and evidence for causation among a broad panel biochemical traits and psychiatric illness. These data could be used to better understand shared genetic architecture between systemic traits and psychiatric disorders, with potential opportunities to leverage these data for translational approaches such as initiating trials to repurpose compounds that modulate the biochemical trait in question. Our sample size was not preselected; we used the largest European ancestry GWAS of each psychiatric trait publicly available at time of analysis, along with a harmonized set of biochemical GWAS from the large UKBB cohort to minimize intercohort heterogeneity. The inclusion criteria for variants in the analyses are described in detail in Materials and Methods and the Supplementary Materials. This study leveraged observational data from GWAS; however, we were able to use the unique properties of genetic variants to facilitate estimates of causal inference.

### Psychiatric GWAS

GWAS summary statistics for nine European ancestry cohorts for the following disorders were obtained from the Psychiatric Genomics Consortium: ADHD (*55*), AN (*56*), ASD (*57*), BIP (*58*), MDD (*59*), OCD (*60*), PTSD (*61*), SZ (*40*), and TS (*62*). Given that cognitive symptoms are pervasively associated with psychiatric illness, we also included a GWAS of general cognitive ability (*63*). Further information regarding these studies is provided in Supplementary Text.

### Blood-based biomarker GWAS

We obtained GWAS summary statistics for a series of blood-based biochemical traits from the large UKBB sample performed by the Neale group (www.nealelab.is/uk-biobank). The key advantage of these data is its large sample size (*N* > 300,000) and that the biochemical traits analyzed were obtained from a single large cohort. Specifically, we use a panel of 50 biochemical GWAS that had high or medium confidence estimates of SNP heritability that were significantly different from zero as outlined in table S1 and Supplementary Text. These traits included lipids, micronutrients, hormones, metabolites, and enzymes.

### Genetic correlation

The genetic correlation between each psychiatric and biochemical trait was estimated using LDSR (*7*), with summary statistic cleaned (“munged”) to contain around one million HapMap 3 SNPs outside the major histocompatibility complex (MHC) with minor allele frequency > 0.05 for consistency (https://github.com/bulik/ldsc). Briefly, LDSR estimates genetic covariance by regressing SNP-wise χ^2^, the product of the marginal SNP effects from both traits (*Z*_1_*Z*_2_), on its LD score, which is an estimate of total LD existing with that SNP. Trait hertiabilities are used to normalize the genetic covariance to obtain genetic correlation (*r*_g_). A key advantage of LDSR is that sample overlap only affects the LDSR intercept and not the slope, meaning that we can accurately estimate *r*_g_ between UKBB biochemical GWAS and psychiatric GWAS with UKBB samples included. We used the Bonferroni method to correct for the 50 traits tested. One biochemical trait was excluded from further analysis, apolipoprotein B, as it exhibited negative heritability within the block jackknifing procedure to estimate the *r*_g_ SE in some instances. The resulting 49 by 10 matrix of LDSR *r*_g_, divided by its SE to obtain *z* score, was subjected to a latent clustering method, finite GMM, with the mclust R package version 5.4.6 (*64*). We selected the most parsimonious clustering configuration based on parametrization of the covariance matrix using the largest Bayesian information criterion value.

### Biochemical polygenic scoring in a severe cognitive deficit subtype of schizophrenia

We sought to further investigate biochemical traits displaying psychiatric genetic correlation and examine their relevance to the clinical dimensions of psychiatric disorders. Specifically, we considered the heterogeneity of cognitive impairment observed in SZ, wherein deficits often manifest before the first psychotic episode and are highly variable in their presentation throughout clinical course (*20*, *65*). We considered biochemical traits that were genetically correlated with either SZ or cognition after the application of multiple testing correction and interrogated their relationship with severe CD in a cohort of SZ cases from the ASRB cohort. The use of these data was approved by the University of Newcastle Human Research Ethics Committee and the ASRB (*20*, *30*, *65*). Previously, Green *et al.* (*20*) used multidimensional GoM clustering with nine cognitive measures to derive subgroups of cognitive performance in the ASRB. The most parsimonious configurations were two clusters of SZ cases termed CD, with more pervasive cognitive impairment, and CS, displaying intermediate cognitive performance relative to CD cases and healthy controls. PGS were constructed for the 25 biochemical traits correlated with either SZ or cognitive ability in a genotyped subset of the ASRB with SZ cases subtyped as CD or CS (*N* = 391; Supplementary Materials). The full details of this cohort and the generation of PGS are described in Supplementary Text. We tested the association of each biochemical PGS with CD status using binomial logistic regression covaried for sex and the first three SNP-derived principal components (Supplementary Materials). The variance explained (Nagelkerke’s *R*^2^) in the full model, with the PGS versus the null (covariates and intercept only) model, was converted to the liability scale, assuming a population prevalence for CD of 0.33% (*67*). The population prevalence for CD is somewhat arbitrary; however, given that the population prevalence of SZ is around 0.7%, and 43% of this portion of the SZ cases in the ASRB cohort was subtyped as CD, we believe that this was an appropriate value to select. A PGS for general cognitive ability and an SZ PRS were also derived in this cohort for comparison (Supplementary Materials).

### LCV models

Genetic correlation may reveal important insights into shared biology between two traits; however, this should not be interpreted as implying a causal relationship in either direction. To evaluate evidence for a causal relationship, we implemented the LCV model to estimate genetic causality between traits, as outlined extensively elsewhere (*9*, *25*, *68*). The LCV framework leverages the bivariate genome-wide distribution of marginal SNP effects on both traits to estimate partial genetic causality. Specifically, the LCV assumes a latent variable, *L*; mediates the genetic correlation between the traits; and tests the strength of the correlation of each trait with *L*. The mixed fourth moments (cokurtosis) of marginal effect sizes for each trait (*Z*_1_, *Z*_2_) are compared to evaluate the proportionality of effects on either trait. The main output of the LCV model is the posterior mean $\hat{\text{GCP}}$, whereby $\hat{\text{GCP}}$ > 0 implies partial genetic causality of trait one on trait two and vice versa. In other words, given $\hat{\text{GCP}}$ > 0, then trait one SNP effects ($Z_{1}^{2}$) tend to be proportionally large on trait two (*Z*_1_*Z*_2_), such that $|E(Z_{1}^{2}\;Z_{1}Z_{2})|\geq |E(Z_{2}^{2}\;Z_{1}Z_{2})|$. As in LDSR, the LDSR intercept is used here to guard against inflation due to sample overlap. We defined partial genetic causality using the recommended threshold of a significantly nonzero |$\hat{\text{GCP}}$| > 0.6, as this was shown by O’Connor and Price in simulations to guard against false positives (*9*). An LCV model was constructed for all genetically correlated psychiatric-biochemical trait pairs. Weak GCP estimates close to zero for genetically correlated traits imply that their relationship is potentially mediated by horizontal pleiotropy, whereby there may be shared genes, but the two traits do not likely exhibit vertical pleiotropy, whereby one trait causally influences another. We also attempted to replicate the observed $\hat{\text{GCP}}$ using a non-UKBB biochemical GWAS (*24*, *26*, *69*, *70*). It should be noted that the posterior mean $\hat{\text{GCP}}$ is not an estimate of the magnitude of any potential causal relationship and should not be interpreted as such, rather it evaluates the strength of evidence for a putative causal relationship using genome-wide SNP effects. The scripts to construct an LCV model are available at https://github.com/lukejoconnor/LCV.

### Mendelian randomization

CRP exhibited strong evidence of partial genetic causality on multiple psychiatric disorders, and, thus, we sought to estimate the magnitude of this causal relationship using univariable MR and MVMR. A detailed description of the MR methodology in this study is provided in the Supplementary Materials, with the theoretical justification for using SNPs as IVs also extensively discussed previously (*8*, *71*). We estimated the total effect of CRP on each disorder using independent genome-wide significant SNPs from a smaller non-UKBB GWAS as sample overlap between exposure and outcome that can bias MR estimates (Supplementary Materials) (*26*). The *F* statistic for IVs for this CRP GWAS was sufficiently strong (*F* > 10), as reported previously (*26*).

Our primary model was an IVW effect estimator with multiplicative random effects, which assumes that all IVs are valid and is less biased by heterogeneity than a fixed-effects IVW estimator (*8*). While the IVW estimator is generally considered the most well-powered approach, the assumption that all IVs are valid is probably unrealistic in practice. As a result, we implemented a series of models that make different underlying assumptions regarding IV validity, specifically, median-based estimators, which assume that most IVs are valid (*72*); a weighted mode estimator and a contamination mixture model, which both assume that, out of groups of IVs having the same asymptotic estimate, the largest group will be composed of valid IVs (plurality valid) (*73*, *74*); and MR-Egger, which includes a nonzero intercept as a test of the average pleiotropic effect and assumes that there is no significant correlation between direct IV effects on the outcome and genetic association of IVs with the exposure (InSIDE assumption) (*75*). As recommended by Bowden *et al.* (*76*), we ensured that the *I*^2^ statistic of IV-exposure effects exceeded 0.9, as this assesses the relative strength of the no-measurement error assumption and, thus, the suitability of using an MR-Egger model. We also tested the effect of using robust regression and penalized estimates for heterogeneity on the IVW, median, and Egger regression estimates (*77*). Evidence of horizontal pleiotropy and outliers were further investigated by testing heterogeneity in the IV/exposure-outcome effects (*78*), performing a leave-one-IV-out analysis (*71*), and testing for a nonzero MR-Egger intercept (*75*) and an MR-PRESSO test of global pleiotropy (also related to heterogeneity) (*79*). Furthermore, we also tested whether there was evidence of a causal effect in the reverse direction (psychiatric disorder as exposure), although MR estimates with binary exposures should be treated cautiously, as described elsewhere (*44*, *80*). Given that only approximately 2,000,000 SNPs were available in the non-UKBB CRP GWAS used, we used the more deeply imputed UKBB CRP GWAS as the outcome here, although these results could therefore be inflated by the sample overlap for AN and MDD.

MVMR was then performed to evaluate the direct effect of CRP on each psychiatric outcome tested when conditioned on BMI and IL-6 signaling, which are both postulated to be closely functionally related to circulating CRP (Supplementary Text) (*81*, *82*). MVMR assumes that IVs are strongly associated with at least one exposure, and, therefore, SNPs were chosen, which were genome-wide significant for at least one phenotype. We constructed an IL-6 and BMI multivariable model separately—that is, CRP conditioned on circulating IL-6 and its receptor (IL-6R), and CRP conditioned on BMI, as well as BMI and IL-6R. The strength of the IVs in each multivariable model was assessed using a two-sample conditional *F* statistic, which tests whether the IVs strongly predict each exposure, conditional on the other exposures in the model (*83*, *84*). When an *F* statistic > 10 could not be achieved, we relaxed the SNP inclusion threshold to represent suggestive significance in the GWAS (*P*_GWAS_ < 1 × 10^−5^; Supplementary Text). We compared direct estimates for each exposure using four different MVMR models—an IVW MVMR estimator, a median-based MVMR estimator, an Egger regression–based MVMR estimator, and a regularization approach whereby least absolute shrinkage and selection operator (LASSO)-type penalization is applied to shrink intercept terms corresponding to IVs predicted as valid (MVMR-LASSO) (*85*). Given that CRP was genetically correlated with several psychiatric GWAS, genetic correlation may result in bias in MR estimates (*9*). However, MR is a valuable extension to the LCV model as it allows for specification of several different assumptions about IV validity and facilitates the estimate of total (univariable MR) and direct (MVMR) effects. The MR analyses were performed using the following packages in R version 3.6.0: TwoSampleMR version 0.5.5 (*86*), MendelianRandomization version 0.5 (*87*), MR-PRESSO version 1.0 (*79*), and MVMR version 0.3 (*83*).

### Genetic overlap between CRP and SZ

We investigated whether any of the lead SNPs (genome-wide significant) reported in the PGC3 SZ GWAS were also associated with CRP (*P* < 5 × 10^−8^). For overlapping genome-wide significant signals, we tested whether there was a shared causal variant or different causal variants underlying these loci, assuming a single causal variant, using the coloc package colocalization method (*88*). Moreover, we used ρ-HESS (https://github.com/huwenboshi/hess) to estimate local genetic covariance between SZ and CRP, as opposed to a genome-wide estimate by LDSR, with local genetic covariance (ρ_g, local_) calculated for munged HapMap 3 SNPs in around 1600 approximately independent LD blocks outside the MHC (*89*, *90*). We derived an estimate of genetic correlation (*r*_g, local_) by dividing ρ_g, local_ by the product of the square roots of CRP and SZ local heritability per LD block, respectively.

### Downstream effects of CRP

We investigated the downstream effect of CRP on circulating levels of 3284 proteins in blood using MR (*91*). We used the larger UKBB CRP GWAS to select IVs to maximize power. The principal MR model was the IVW estimator with multiplicative random effects to maximize power. Proteins that demonstrated at least nominal association with CRP levels after multiple testing correction (FDR < 0.1) were investigated for protein-protein interaction and overrepresentation in biological pathways using STRING version 11.0 (*92*).

### Statistical analysis

All statistical analyses used in this study were fully described in the previous sections, with additional information as necessary in the Supplementary Materials.

## Supplementary Materials

### This PDF file includes:

Psychiatric genome-wide association studies Blood-based biomarker GWAS in the UK biobank (Neale group) Description of the Australian Schizophrenia Research Bank (ASRB) cohort Medelian randomisation methodology Model violations in the latent causal variable (LCV) approach Colocalisation analyses between schizophrenia and C-reactive protein Figs. S1 to S3

## Notes

View/request a protocol for this paper from *Bio-protocol*.
